# Informing real-world practice with real-world evidence: the value of PRECIS-2

**DOI:** 10.1186/s12916-018-1071-1

**Published:** 2018-05-21

**Authors:** Gila Neta, Karin E. Johnson

**Affiliations:** 1 Rockville, MD USA; 2 Seattle, WA USA

**Keywords:** Pragmatic trials, Explanatory trials, Real-world evidence, Effectiveness, Usual clinical practice, PRECIS-2

## Abstract

Real-world evidence is needed to inform real-world practice. Pragmatic controlled trials are intended to provide such evidence by assessing the effectiveness of medicines and other interventions in real-world settings, as opposed to explanatory trials that assess efficacy in highly controlled settings. Dal-Ré and colleagues (BMC Med 16:49, 2018) recently performed a literature review of studies published between 2014 and 2017 to assess the degree to which studies that self-identified as pragmatic were truly so. The authors found that over one-third of randomized controlled trials of drugs and biologics that were self-labeled as pragmatic used placebo controls (as opposed to usual care), tested medicines before licensing, or were conducted in a single site. Further, they proposed that, in order to improve the reliability of the ‘pragmatic’ label, investigators should assess their trials using the PRECIS-2 tool upon submission to funders, ethics boards, or journals. We appreciate the value of PRECIS-2 as an indicator to assess the pragmatic versus explanatory features in a trial, and we herein highlight the potential challenges and opportunities that may arise with its systematic and widespread use.

## Background

The term ‘pragmatic controlled trial’ (PCT) was first coined in 1967 by Schwartz and Lellouch [1], and is broadly defined as a randomized controlled trial (RCT) whose purpose is to inform decisions about clinical practice. PCTs assess the effectiveness of an intervention “*under practical conditions*,” maximizing external validity by studying interventions in the context of routine clinical practice conditions [2]. In contrast, explanatory trials measure efficacy and thus prioritize internal validity. While critical for demonstrating the efficacy and safety of medicines, explanatory trials can be poor predictors of real-world effectiveness. The main advantage of PCTs is the fact that they are primarily designed to answer decision-making questions about real-world applicability and generalizability, for example, by comparing the effectiveness of two treatment approaches among heterogeneous patients within the context of real-world practice, whilst using outcomes that matter to end users, including physicians, patients, and administrators [3–5].

The distinction between an explanatory trial and a PCT is not dichotomous, but can be viewed on a continuum and along a variety of dimensions [5]. With this in mind, a CONSORT working group on PCTs developed a tool, called the Pragmatic-Explanatory Continuum Indicator Summary (PRECIS), to help trialists make study design decisions suited to their intended purpose [6]. After initial use, the PRECIS tool was further refined and validated (PRECIS-2) [5], mapping nine domains of a trial onto a wheel, where each spoke represents a feature of the trial that can be characterized on an explanatory–pragmatic spectrum. The nine domains of the tool address patient eligibility and recruitment; study setting; features of how the intervention is delivered, including organizational resources requirements, flexibility in delivery protocol and adherence assessments, and the closeness of follow-up; patient-centeredness of the primary outcome; and the extent to which all data are included in the primary analyses [7]. The PRECIS-2 tool has been used for both trial design and assessment [5, 8, 9].

Dal-Ré et al. [10] recently reviewed published literature between 2014 and 2017 to assess the degree to which studies that self-identified as pragmatic are truly so. They found that over one-third of RCTs of drugs and biologics that were self-labeled as pragmatic used design features viewed as incongruent with the pragmatic approach such as placebo controls (as opposed to usual care), investigational medicines, or single-site settings. The authors proposed that, in order to improve the reliability of the ‘pragmatic’ label, investigators should assess their trials using PRECIS-2 upon submission to funders, ethics boards, or journals, and should include ratings with explanations and supporting documentation. We agree that there is a need for systematic consideration of external validity, and we appreciate the value of PRECIS-2 as one such indicator. Herein, we draw attention to the potential challenges and opportunities that may arise with the systematic and widespread use of PRECIS-2.

## Challenges and opportunities

As Dal-Ré et al. indicate [10], there is increasing interest in real-world evidence, especially that which is generated from high-quality PCTs. This interest is due, in part, to a ‘voltage drop’ in effectiveness as interventions tested in explanatory trials move into real-world situations [11]. Further, given the emphasis of PCTs on generating information that is salient to decision-making, this type of study can provide insights about outcomes, such as cost or quality of life in usual care, to fill the gap remaining after market approval and to fully guide decisions by patients, physicians, and policymakers in selecting the optimal treatment [12, 13].

Ensuring fit-for-purpose is critical to effective interpretation of evidence. Dal-Ré et al.’s [10] suggestion to provide PRECIS-2 ratings and supporting documents could help decision-makers assess the degree to which study proposals and research findings are appropriately characterized. Further, detailed information on how and why studies are pragmatic in different domains helps to understand the applicability of evidence in particular settings and patient populations, for example, such as when a clinic is deciding to implement a new finding in their practice [14].

Nevertheless, caution should be exercised regarding the proposal. The PRECIS-2 tool is designed to be used by a group and to foster discussion about study features. In a previous study [9], we used the PRECIS-2 tool to assess five PCTs. Raters often struggled to use the tool, and large differences in inter-rater reliability were observed despite having access to detailed study information and common training. In follow-up conversations, different perspectives on various aspects, such as what constitutes ‘usual care’, emerged. Similarly, Bratton et al. [15] described how, throughout the development of the BLISTER trial, team members debated whether the trial was pragmatic or explanatory; despite having used the PRECIS wheel, the team needed extra guidance and scores ranged widely on several domains. The authors also highlighted the value of discussion, not just the mean score, to reach a greater consensus on the degree of pragmatism.

Absolute ratings are thus challenging when using PRECIS-2 and, even when accompanied by an explanation and supporting documentation, may be difficult for use by reviewers evaluating journal, protocol, or grant submissions. This is not a criticism of the usability of PRECIS-2, but rather a comment on the challenges of evaluating external validity. Even when the trial team self-rates their study, which we agree is more appropriate than an independent external evaluation, the use of rating information is complicated. For grant review, reviewers would be required to understand how to use and interpret the ratings and consider if PRECIS-2 is in fact the best tool. For peer-review publishing, an additional challenge emerges in that there is no clear or consensus threshold for what should be labeled as pragmatic.

One example of the complexity of the PRECIS-2 tool is the issue of a single-center trial, which, as Dal-Ré et al. indicate [10], would be likely to receive a more explanatory rating. However, if the trial is designed to address a question such as, for example, patient satisfaction with medication infusion timing, which typically occurs in such a setting, it could be pragmatic. This sort of nuance might be lost among the volume of details assessed by a busy grant reviewer. The investigator could justify giving themselves a rating of 3 instead of 1 on the setting domain by, for example, explaining that their intervention is typically performed in a specialized setting represented by their single center. The reviewer, in turn, would need to understand this potential complexity to decide whether they agreed with the pragmatic assessment.

Another example of the complexity of applying the PRECIS-2 tool is the value of capturing trial changes over time. Given that a trial design may change from initial concept to execution, the degree of pragmatism may change. Assessing the degree of pragmatism at one time point could miss valuable insights about how the intervention had to be adapted to better fit a specific setting. For example, constraints in a healthcare system where an intervention is being tested may result in changes to flexibility in delivery or adherence. The circumstances that led to these changes could be important to capture for end users. The PRECIS-2 criteria can be used to assess pragmatic elements at multiple time points and across different settings, but cannot in isolation capture critical information on adaptations and how an intervention could work in broader settings given these adaptations [16].

## Conclusions

The PRECIS-2 tool can be a valuable instrument to characterize the applicability of a trial and enhance the reliability of the ‘pragmatic’ label. However, we caution that the tool may be limited in its interpretation and its widespread application would require ongoing training in using the tool for both trialists and reviewers evaluating journal, protocol, or grant submissions. Nevertheless, PRECIS-2 could enhance transparency and accountability in the reporting of RCTs and ensure that real-world evidence appropriately incorporates real-world treatment patterns and decision-making needs.
